# Completion of an Experiment

**DOI:** 10.1128/mSphere.00678-18

**Published:** 2018-12-19

**Authors:** Michael J. Imperiale, Ira Blader, Patricia Bradford, Sarah D'Orazio, W. Paul Duprex, Craig D. Ellermeier, Ana Fernandez-Sesma, Katherine McMahon, Aaron Mitchell, Marcela F. Pasetti, Susannah Tringe

**Affiliations:** a University of Michigan Medical School, Ann Arbor, Michigan, USA; b University at Buffalo, Buffalo, New York, USA; c Antimicrobial Development Specialists, LLC, Nyack, New York, USA; d University of Kentucky, Lexington, Kentucky, USA; e University of Pittsburgh School of Medicine, Pittsburgh, Pennsylvania, USA; f University of Iowa, Iowa City, Iowa, USA; g Icahn School of Medicine at Mount Sinai, New York, New York, USA; h University of Wisconsin—Madison, Madison, Wisconsin, USA; i Carnegie Mellon University, Pittsburgh, Pennsylvania, USA; j University of Maryland School of Medicine, Baltimore, Maryland, USA; k Department of Energy, Joint Genome Institute, Walnut Creek, California, USA

## EDITORIAL

At the beginning of 2017, *mSphere* initiated an experiment in peer review called mSphereDirect (1). At that time, we sensed the desire among authors to have more control over the peer review process, which had become slow and rigid, with some even arguing that peer review is “broken.” mSphereDirect was our attempt to cede control of the process to authors by putting them in charge of soliciting their own manuscript reviews, revising their manuscripts according to those reviews, and submitting them directly to the journal. At that point, the journal would make an accept or reject decision within 5 business days. In one way, we viewed this as a democratization of the author-solicited review processes used by American Academy of Microbiology members with *mBio* or by National Academy of Sciences members with *Proceedings of the National Academy of Sciences*. We assumed that authors would know who is best qualified to review their work and could also expedite the process. We developed rigorous guidelines, by specifying strict reviewer qualifications and processes, to try to ensure that the system would not be gamed.

As of 30 November 2018, *mSphere* had received 71 submissions via the mSphereDirect pathway. This represented 7.2% of the total submissions to the journal during the same time period. The acceptance rates for mSphereDirect and for regular *mSphere* submissions were 72.6% and 45.0%, respectively. Overall, while we achieved the intended result of having mSphereDirect manuscripts accepted at a higher rate since the peer review process preceded submission, the number of these manuscripts has been only a small fraction of the total submissions to the journal.

In addition to these quantitative data, our experience has been that the process did not work as smoothly as we had hoped. A number of issues and concerns surfaced as we conducted the experiment. First, we heard that authors felt awkward asking colleagues to review their manuscripts, and conversely, some scientists were uncomfortable serving as nonblind reviewers. As such, one could argue that mSphereDirect was not as democratic as it might seem because it favored those whose comfort level was the highest. Second, we noticed that many of the reviews (a significantly larger percentage than that of the reviews that we ourselves solicited as editors for regular submissions) seemed to be somewhat superficial and uncritical. Not always knowing the reviewers, it was hard for us to know whether the work was truly as good as the reviews stated or whether authors had chosen colleagues whom they knew would not criticize their work. Tasked with maintaining our editorial standards, we sometimes rejected manuscripts from authors who technically followed all the rules because we were less than certain about the significance of the work. Despite our trying to improve the process by periodically modifying the guidelines, we found ourselves having the same types of doubts and often having to review the manuscripts ourselves. Needless to say, these rejections sometimes led to hard feelings. In some instances, it was felt that the manuscripts could benefit from further improvement, yet the need for a yes or no decision did not allow for additional revision.

At our annual Senior Editors meeting in October, we had a long discussion about mSphereDirect, followed by a series of e-mail exchanges. We talked about the goals of the experiment, our experiences, and whether it was worth continuing. At the end of the day, we have decided to discontinue the mSphereDirect submission pathway. We will no longer accept mSphereDirect submissions after 28 February 2019. Authors who began the process (i.e., contacted potential reviewers) prior to today (19 December 2018) but are unable to meet the 28 February deadline should contact *mSphere* staff as soon as possible (mSphere@asmusa.org).

We express our thanks to Tom Shenk and Pat Schloss, the former and current Chairs of the ASM Journals Committee, respectively; Barbara Goldman and Melissa Junior, the former and current Directors of ASM Journals, respectively; Stefano Bertuzzi, ASM CEO; and the *mSphere* staff (Jasmine Wallace, Noel Lin, and Amanda Donaldson) for their support and efforts over the past 2 years. Mostly, however, we thank the many authors and reviewers who participated in this experiment. It is our goal to continue to innovate at *mSphere*: please stay tuned!
